# Randomized controlled trial reporting guidelines should be updated to include information on subsequent treatments

**DOI:** 10.1002/cncr.35922

**Published:** 2025-05-29

**Authors:** Dawn Lee, G. J. Melendez Torres

**Affiliations:** ^1^ PenTAG University of Exeter Medical School St Luke's Campus Exeter UK

**Keywords:** cost‐effectiveness, PFS2, reporting guidelines, subsequent treatment, surrogacy, time to next treatment

## Abstract

This paper highlights the urgent need to update randomized controlled trial reporting guidelines to include consistent and detailed data on subsequent treatments, particularly in oncology, where postprogression therapies significantly influence overall survival and cost‐effectiveness assessments. Drawing on our experience as an External Assessment Group and National Institute for Health and Care Excellence Committee members, a systematic review of renal cell carcinoma trials and broader regulatory guidance, we advocate for standardized reporting of subsequent treatment types, timing, and durations to enhance the reliability of health technology assessments and support more informed decision‐making.

## RANDOMIZED CONTROLLED TRIAL REPORTING GUIDELINES SHOULD BE UPDATED TO INCLUDE INFORMATION ON SUBSEQUENT TREATMENTS

As oncology trials move into earlier lines of treatment and treatment pathways differ across countries, it is increasingly important to understand the subsequent treatments received. This is important both for interpreting data on overall survival and for calculating cost‐effectiveness. Even when a trial protocol mandates that patients should have the same treatments after progression, patients receiving the more effective treatment will receive treatment earlier, leading to a different effect. Additionally, the treatments available in later lines are often influenced by the therapies used initially, as using treatments with similar mechanisms of action in sequence is unlikely to yield strong efficacy.

Unfortunately, data on trial protocol rules in relation to subsequent treatments and the actual treatment mix and time to next treatment in trials are not consistently reported at present.

During our recent systematic review of clinical evidence to inform National Institute for Health and Care Excellence’s (NICE’s) pathways pilot appraisal in renal cell carcinoma. We identified 26 relevant randomized controlled trials (RCTs). Four of the 26 trials reported no information on subsequent treatments, including one trial reported as recently as 2021.^1^ Where subsequent treatment was reported, it was generally contained in supplementary information, and the format of the information varied considerably. Only one study reported the time to next treatment. This is line with the findings of a recent assessment of the reporting of post‐progression treatment in cancer trials leading to Food and Drug Administration approval and RCTs published between 2018 and 2020.^2^ This is important as in renal cell carcinoma (as in many cancers) progression‐free survival was found to be a poor surrogate for overall survival because of the impact of subsequent treatments.^3^, ^4^

A key component of the cost‐effectiveness case for many oncology treatments is the cost and effectiveness impact of subsequent lines of therapy.^5^ Generalizability of trial data to real‐world practice cannot be adequately assessed without access to data on what subsequent treatments have been received. The costs of the full treatment pathway cannot be calculated and attempts to account for differences in subsequent therapies across trial cannot be made without access to consistent data.

The European Medicines Agency recommends not only the collection of data on the next line of therapy received but also that the time on next‐line therapy is captured in most studies.^6^ Where lack of efficacy of further treatments might be a concern, PFS2 should be collected. PFS2 is defined by the European Medicines Agency as the time from randomization to objective tumor progression on next‐line treatment or death from any cause. In some cases, time to next line of therapy may be used as proxy for PFS within health technology assessment. If PFS2 and time to next treatment data were consistently reported, the ability to conduct and assess source of bias within subsequent indirect treatment comparisons, cost‐effectiveness analysis and health, technology assessments would be considerably increased.

Our experience as NICE External Assessment Group and Committee members is that reporting of data for subsequent treatments continues to be poor. We are therefore encouraging manufacturers to improve trial reporting in future to align with the changes recently implemented to the NICE Single Technology Appraisal template to better capture these data.^7^

To be of most use when later health technology assessment, the following details are required:

- Information on what subsequent treatment data were collected within the trial protocol; for example, how many lines of treatment were considered and whether subsequent treatment was collected by individual drug or by broad class
- Reporting of subsequent treatments received according to the line of treatment. At minimum, this should include the next line of treatment. Where a reasonable proportion of patients are expected receive more than one subsequent line of treatment within trial this should include all lines received
- Reporting of subsequent treatments received including the number and percent of patients who received such treatment from both the intent‐to‐treat population and those eligible to receive a subsequent treatment (for example, those who have progressed and are alive on progression)
- Reporting of subsequent treatments used in combination as a combination treatment rather than as individual components
- Reporting of both drug and nondrug subsequent treatments received (e.g., stem cell transplants, surgery, radiotherapy)
- Reporting of the mean time (e.g., restricted mean survival time) spent on each subsequent treatment received including a measure of uncertainty such as the 95% CI
- Reporting of time to next treatment and PFS2 data where these have been collected with associated Kaplan–Meier plots

## AUTHOR CONTRIBUTIONS

**Dawn Lee**: Conceptualization; methodology; writing — original draft. **G. J. Melendez Torres**: Writing — review and editing; methodology; validation; supervision.

## CONFLICT OF INTEREST STATEMENT

Both Dawn Lee and G. J. Melendez‐Torres are employees of the University of Exeter, which receives a grant from the NIHR to conduct evaluations as an external assessment group for NICE.

## Data Availability

Data sharing is not applicable to this article as no new data were created or analyzed in this study. For the purpose of open access, the author has applied a Creative Commons Attribution (CC BY) licence to any Author Accepted Manuscript version arising from this submission.
